# Impact of interventions addressing perinatal mental health on loneliness and/or satisfaction with social support: systematic review

**DOI:** 10.1186/s12888-026-08035-8

**Published:** 2026-04-11

**Authors:** Cristina Vasquez, Apphia Ruth D’souza, Rebecca Nowland, Billie Lever Taylor, Katherine Adlington, Eiluned Pearce, Alexandra Pitman

**Affiliations:** 1 UCL Division of Psychiatry, University College London, London, UK; 2 South London and Maudsley NHS Foundation Trust, London, UK; 3 North London NHS Foundation Trust, London, UK; 4 School of Nursing and Midwifery, University of Lancashire, Preston, UK; 5 Manchester Institute of Education, University of Manchester, Manchester, UK; 6 Florence Nightingale Faculty of Nursing, Midwifery & Palliative Care, Kings College London, London, UK; 7 Section of Women’s Mental Health, Institute of Psychiatry, Psychology and Neuroscience, Kings College London, London, UK; 8 East London NHS Foundation Trust, London, UK; 9 Oxford Health NHS Foundation Trust, Oxford, UK; 10 The Oxford Institute of Clinical Psychology Training and Research, University of Oxford, Oxford, UK

**Keywords:** Perinatal care, Mental health, Anxiety, Depression, Loneliness, Social support, Systematic review

## Abstract

**Objectives:**

Loneliness and low social support are associated with poor mental health in the perinatal period (pregnancy and one year postnatal). However, no reviews have explored systematically whether interventions aimed at improving perinatal mental health also improve loneliness and/or social support satisfaction (SSS). We aimed to address this gap in the literature, to improve our understanding of mechanisms underlying interventions addressing perinatal mental health and loneliness and/or SSS.

**Methods:**

We conducted a systematic review of studies evaluating the effectiveness of interventions to address perinatal mental health and also measuring either loneliness or SSS. We searched six electronic databases and eight grey literature sources. Two reviewers independently screened papers for eligibility and assessed risk of bias. Findings were presented as a narrative synthesis.

**Results:**

Of 6,422 unique retrieved records, we included 26 eligible studies (measuring both mental health and either loneliness or SSS). Of these, four studies measured mental health and loneliness, 21 measured mental health and SSS, and one measured mental health and both loneliness and SSS. Only eight of the 26 included studies were deemed to present low risk of bias. Of 17 included randomised controlled trials (RCTs), six identified significant improvements in loneliness/SSS compared to controls, but only three of these also identified significant improvements in anxiety and/or depression. Interventions with some evidence of effectiveness in improving loneliness/SSS were those facilitated by professionals, whilst those with some evidence of effectiveness in improving mental health were those that involved peer contact. Only one study conducted formal mediation analysis to delineate the pathways between these outcomes, finding no support for their hypothesis that an improvement in SSS mediates a reduction in depression.

**Conclusion:**

We found evidence that some (but not all) interventions that address perinatal mental health also improve loneliness and/or SSS. We found little empirical work to explain the pathways between these variables. Our findings suggest the need for further work exploring the relationships between loneliness/SSS and mental ill-health among perinatal parents. This will identify opportunities to prevent the onset or worsening of mental ill-health in parents (and their children) at risk of mental health problems.

**Supplementary Information:**

The online version contains supplementary material available at 10.1186/s12888-026-08035-8.

## Background

The perinatal period (encompassing pregnancy and one year postnatally) carries an increased risk of depression, with an estimated 15–65% of pregnant women experiencing antenatal depression [16] and one in five women experiencing postnatal depression [57]. Similarly, prevalence of any anxiety disorder is estimated to affect one in five perinatal women in the general population [20]; and 17% of women with a history of bipolar disorder are likely to relapse experiencing severe episodes in the postnatal period [59]. The prevalence of perinatal anxiety in men ranges from 3.4% to 25% during the antenatal period, and from 2.4% to 51.0% during the postnatal period [43].

Perinatal psychiatric disorders can impact parent-infant interactions, with severe physical and emotional consequences for postnatal parents and their children [24]. There is growing evidence supporting the reciprocal relationship between loneliness and depression among parents, with impacts on children [40] and of pregnancy as a potential preventive opportunity for postnatal depression [25]. Potential risk factors for mental ill-health in the perinatal period are loneliness and dissatisfaction with social support [38], suggesting that these social factors may be viable intervention targets. Similarly, a recent systematic review and meta-analysis [6] identified significant correlations between low social support and the risk of depression and anxiety in pregnant women.

A scoping review [29] identified that around 32–42% of perinatal women report loneliness, with estimates as high as 100% for specific populations of perinatal parents (e.g. gender minority parents, or parents of children with specific health conditions, etc.). This compares with loneliness estimates of around 3–12% among young and middle-aged men and women in European countries [56]. Considering the established longitudinal association between loneliness and depression and anxiety in the wider population [36] and a time-sensitive window in which to treat perinatal difficulties, it is crucial to identify interventions focused on social support that might address perinatal mental disorders.

Loneliness describes the experience in which a person perceives the quality of their relationships as inadequate compared with those they desire [41]. It is related to other subjective aspects of social relationships, but particularly to perceived social support: the quality of practical and emotional support an individual believes they have available to them [58]. While some authors have defined perceived social support as the subjective evaluation of the availability of support [12], others add to this concept the degree of satisfaction with this social support [48]. ‘Social support satisfaction’ (SSS) describes whether an individual’s social relationships and perceived support match those they desire. Therefore, loneliness and SSS are similar concepts reflecting a mismatch between an individual’s actual experience of social relationships and their expectations. In this review, both these subjective aspects of dissatisfaction with social relationships were treated as adjacent to remain comprehensive and also to attempt to understand the relationship between them.

Interventions targeting loneliness in populations with mental health problems, but not specifically in the perinatal period, are broadly classified into ‘direct interventions’ (to reduce loneliness or enhance social support specifically) or ‘indirect interventions’ (addressing broader wellbeing approaches that could impact loneliness) [35]. Direct interventions are sub-classified into psychological interventions intended to change maladaptive cognitions, social skills training, supported socialisation, and wider community approaches to promote integration [35]. Of these, the evidence suggests that changing cognitions is the most promising for alleviating loneliness among people with mental health problems, but more robust evidence is needed [35]. The acceptability of interventions is also critical, and this is likely to vary in the perinatal context by formal (organisations and healthcare specialists, e.g. hospitals, midwives) and informal (individuals, e.g. relatives, friends) support sources, and by whether support is psychological, instrumental, educational or informational [1]. For example, survey evidence suggests that postpartum women show lower levels of satisfaction with formal social support than informal sources of support [1].

It is possible that some interventions addressing perinatal mental health might also have indirect effects on loneliness and/or SSS (or indeed in some cases, direct effects). Understanding the impact of perinatal mental health interventions on experiences of loneliness and social support among perinatal parents is important because interventions that achieve both these goals have the potential for substantial public health impact for a defined population in a unique transitional phase. This might be realised in terms of longer-term positive impacts on parental and child mental health and social functioning [24, 25, 40]. It is also important to understand the mechanisms of change in interventions of this kind, for example the temporal nature of changes in social and mental health outcomes. To our knowledge, no systematic reviews have examined the effectiveness of interventions aiming to improve perinatal mental health that also measure loneliness and/or SSS. We aimed to address this gap in the literature and contribute to an understanding of mechanisms and potential improvements in perinatal mental healthcare.

## Methods

We conducted a systematic review of studies evaluating the effectiveness of interventions to address perinatal mental health and also measuring either loneliness or SSS. We followed the Preferred Reporting Items for Systematic Reviews and Meta-Analyses (PRISMA) guidelines and pre-registered our protocol on the PROSPERO database (CRD42021253688).

### Eligibility criteria

Our review included studies that met the eligibility criteria listed in Table 1, using the PICOS approach. We limited the search to full-text studies published in English or Spanish, with no restrictions regarding publication date.

**Table 1:** Eligibility criteria using the PICOS approach

PICOS	Eligibility criteria
Population	Parents at any stage of the perinatal period (from pregnancy up to 12 months after giving birth or in the context of a miscarriage or stillbirth). There were no restrictions regarding parents’ gender, age or nature of parental relationship to offspring (e.g., genetic, adoptive, step, partner of pregnant individual, etc.).
Intervention	Interventions addressing any mental health symptom/diagnosis in the perinatal period, with no restrictions on format (remote, in-person, individual, group, digital). Studies exclusively targeting substance use/dependence but with no mental health symptom measurement (i.e., lacking a measure of anxiety, depression, or other aspect of mental wellbeing) were excluded^a^. We did not stipulate that included studies had to state an explicit intention of the intervention to improve loneliness or social support as a means of improving participants’ mental health or conduct a formal mediation analysis involving these variables. Given our criteria for eligible outcomes (i.e. mental health *and* either loneliness or social support satisfaction; SSS), we assumed that any evaluation of an intervention designed to improve perinatal mental health that also measured these outcomes was presumed have a positive influence on both (and/or that the intervention would involve loneliness or SSS in its mechanistic pathways).
Comparisons	Studies that either included a control group (exposed to another intervention or treatment as usual) or used pre/post comparison for single groups were included.
Outcomes	a) Subjective aspects of social relationships: A quantitative self-reported measure of two specific subjective aspects of social relationships as outcome(s) was required for inclusion: either loneliness or social support satisfaction (SSS). Measures to capture these might include the UCLA Loneliness Scale [47] or the Social Support Questionnaire [48] and could be either a validated or unvalidated measure^b^. Only measures assessing the subjective experience of having/lacking adequate emotional and/or instrumental social support were eligible as SSS measures. Studies where individuals self-reported how satisfied they were with the support provided exclusively by one specific person or specific group (e.g., partner or intervention facilitators) were excluded as these did not ascertain perceptions of total support in omitting the context of other support providers. When measures included multiple dimensions of social support, only the subscale relevant to SSS was considered. AND b) Mental health: A quantitative measure of any mental health symptom as outcome(s) was required for inclusion. This included (but was not limited to) general mental wellbeing, maternal psychopathology, depression, anxiety, stress, post-traumatic stress disorder, and features of personality disorder. Again, these could be validated measures, such as the Edinburgh Postnatal Depression Scale [15] for measuring depression, or unvalidated measures ^b^.
Study designs	Randomised controlled trials (RCT), non-randomised controlled trials and single group studies with pre/post comparison were included. Cohort studies were also eligible when they used longitudinal data to simulate potential intervention effects of exposure to interventions.

^a^ We did not exclude studies where perinatal parents had comorbid drug/alcohol difficulties

^b^ In our original protocol we planned to only include studies that used validated outcome measures. However, due to finding a number of studies at screening that used an unvalidated measure we revised our protocol to include studies using either validated or unvalidated measures to be more comprehensive

### Search strategy

We searched six electronic databases and eight sources of grey literature between May and July 2021, updated on 18th September 2023. We used free text terms and MeSH terms, such as “perinatal”, “mental* health”, “interven*” and “lonel*”, for each of these databases following the PICOS approach (Supplementary Table 1).

### Study selection

We imported records to a systematic review software package (EPPI-Reviewer) and deduplicated (automatically using an 86% similarity threshold, and then manually). One researcher screened all deduplicated records on title and abstract, and another researcher independently screened a randomly selected 10% of these records. Two researchers independently screened all full text records. Inter-rater reliability was assessed using an online Cohen’s kappa calculator (https://idostatistics.com/cohen-kappa-free-calculator/). Disagreements were discussed first between the two screeners, and then with other team members when needed to reach consensus. To assess the eligibility based on outcomes, we examined the construct that each loneliness or SSS scale used, regardless of the name given to this variable, and included those that matched the definitions described above.

### Data extraction

We extracted data from included studies using an adaptation of the Cochrane Data Extraction Form capturing: study design, methods, intervention characteristics, and findings related to loneliness/SSS and mental health.

### Risk of bias

We assessed risk of bias using the Cochrane Risk-of-Bias (RoB2) for RCTs [9, 54] or the ROBINS-I for non-randomised and single-group designs [8, 53]. Two researchers independently assessed risk of bias for all selected studies. Discrepancies were resolved through discussions. We also tested for publication bias using Egger’s Test [19].

### Data analysis

We planned to conduct a meta-analysis to synthesise quantitative findings unless high heterogeneity indicated that a narrative synthesis was appropriate [44]. Heterogeneity was assessed by tabulating and comparing study design, subpopulations (pre- and post-natal; sex), intervention components, and outcome measures.

In the case of high heterogeneity, we aimed to follow the Synthesis Without Meta-analysis (SWiM) guidelines [10] and to synthesise the results by the social outcomes measured (loneliness or SSS) as the main focus for our systematic review. We also aimed to group studies by their design (i.e., RCT vs. non-RCT) to improve methodological comparability. The primary metric for cross-study comparison was the presence or absence of a positive direction of effect (i.e., significant improvement) in the social outcome measure for the treatment group, involving vote counting as the synthesis method.

We also aimed to report findings for loneliness and SSS as distinct constructs, attempting (where they were investigated together) to understand relationships between them.

In our synthesis we considered our risk of bias assessments and the number of studies with consistent findings when assessing the certainty of our synthesis.

Data were presented using tables and narrative synthesis.

## Results

### Studies identified

**Fig. 1:**
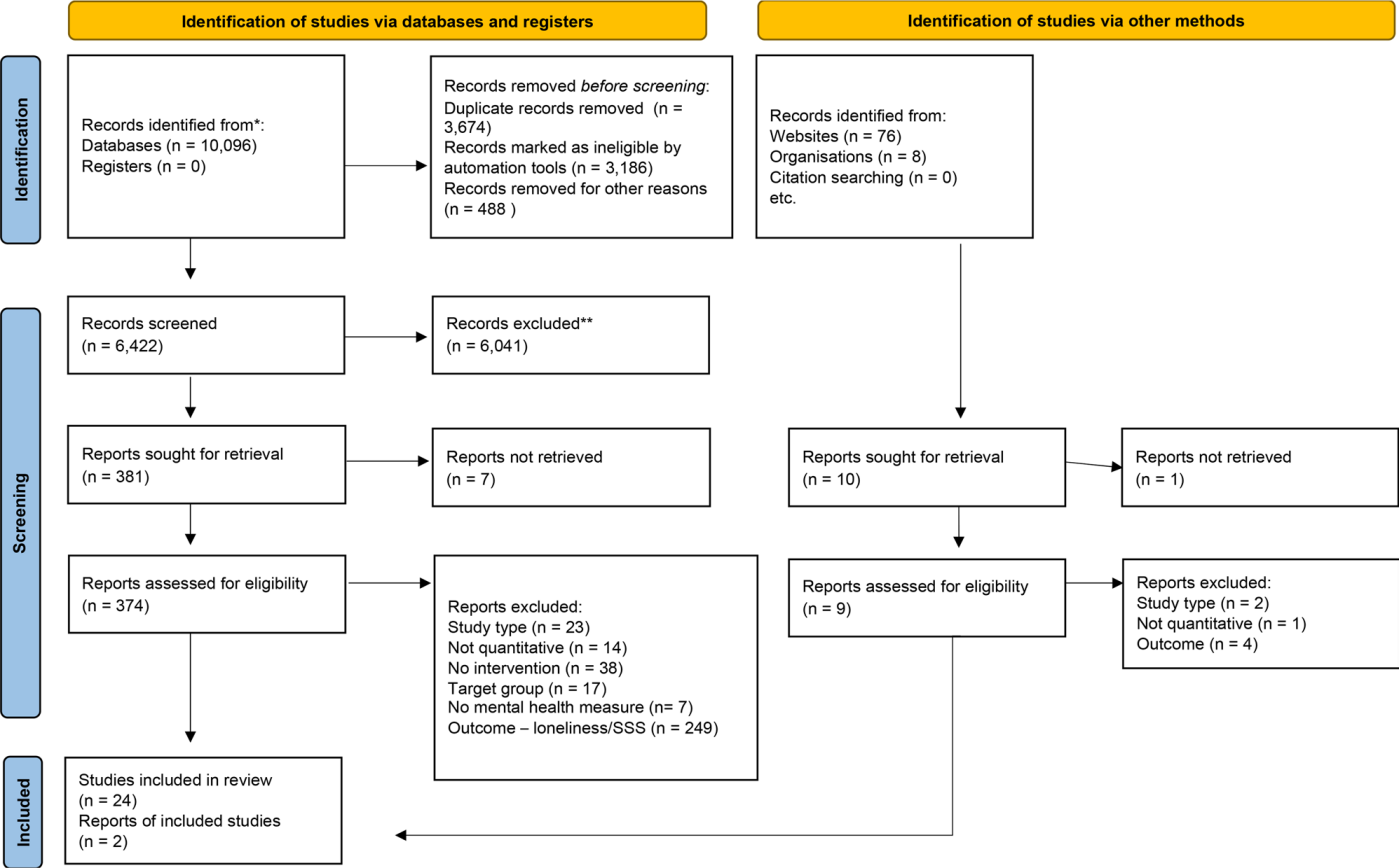
Prisma flow diagram describing studies’ selection. Source: Diagram created using PRISMA2020 [23]

We screened a total of 6,422 unique records from electronic databases and 84 records from grey literature sources on title and abstract. We identified that loneliness and SSS were assessed using a variety of measures across studies; authors verified that measures used in every included study met the definition and inclusion criteria outlined in our protocol. We achieved a moderate level of agreement for both the title/abstract screening (Cohen’s kappa = 0.49; agreement = 92.4%), and full-text screening (Cohen’s kappa = 0.57; agreement = 80.5%). We identified 26 eligible papers (Fig. 1): 23 identified through the 2021 searches and 3 from the 2023 searches [2, 42, 51].

### Risk of bias

The quality of studies was judged to be poor overall, with only 31% of 26 studies judged to be at low risk of bias. Details are tabulated under the sub-categories below. Key methodological issues noted were inappropriate statistical analyses, confounding and missing outcome data. Only three studies presented effect measures with standard errors (Supplementary Table 2), so we were only able to conduct Egger’s test [19] based on three values (Supplementary Fig. 1). This was therefore difficult to interpret [55]. We noted that 7 of 26 studies reported positive findings for loneliness or SSS outcomes and 11 of 26 studies reported positive findings for mental health outcomes, so we considered publication bias to be unlikely.

### Data synthesis

Given the high degree of heterogeneity regarding interventions (different aims, facilitators, delivery formats) and outcomes (Table 2), we conducted a narrative synthesis following SWiM guidelines [10]. We grouped the studies according to their design (RCT *versus* non-RCT) and the social outcomes measured (loneliness; SSS) alongside mental health outcomes. For all these studies we identified whether each study presented a positive direction of effect for the social outcome (loneliness or SSS) and commented on whether the mental health intervention appeared to take a direct or indirect approach to addressing loneliness or SSS; we also commented on risk of bias and estimated certainty of evidence.

**Table 2:** Summary characteristics of the included studies (for detailed characteristics see Tables 4, 6 and 8)

Variable		RCTs assessing loneliness (*N* = 5)		RCTs assessing SSS only (*N* = 12)		Non-RCTs (all assessed SSS; *N* = 9)		All studies (*N* = 26)	
		N	%	N	%	N	%	N	%
Study design	RCT	5	100%	12	100%	0	0%	17	65%
	Non-randomised controlled trial	0	0%	0	0%	4	44%	4	15%
	One group - controlled before and after	0	0%	0	0%	4	44%	4	15%
	Cohort-study	0	0%	0	0%	1	11%	1	4%
Location of intervention	High-income country	5	100%	11	92%	7	78%	23	88%
	Middle- or low-income country	0	0%	1	8%	2	22%	3	12%
Sex of participants	Female	5	100%	9	75%	9	100%	23	88%
	Parents in dyads (heterosexual couples)	0	0%	3	25%	0	0%	3	12%
	Parents in dyads (same sex couples)	0	0%	0	0%	0	0%	0	0%
Perinatal period	Pregnant	1	20%	4	33%	1	11%	6	23%
	Postnatal	4	80%	4	33%	3	33%	11	42%
	Pregnant and postnatal	0	0%	4	33%	5	56%	9	35%
Marital status	Single mothers	0	0%	2	17%	1	11%	3	13%
	Married / cohabiting couples	3	100%	3	25%	2	22%	8	33%
	No predominant marital status	0	0%	7	58%	6	67%	13	54%
Sample’s predominant demographic characteristics^a^	Socioeconomically vulnerable (ethnic minority, low income, and/or refugees)	0	0%	6	50%	5	67%	11	42%
	Vulnerable to or had mental health problems	4	80%	3	25%	4	44%	10	38%
	Previous perinatal problems	1	20%	2	17%	0	0%	3	12%
Type of intervention	Direct interventions	1	20%	4	33%	5	56%	11	42%
	Indirect interventions	4	100%	8	67%	4	44%	15	58%
Components of intervention^a^	Social skills training	0	0%	2	17%	3	33%	5	19%
	Support for developing social interactions	1	20%	3	25%	2	22%	6	23%
	Parenting skills	0	0%	7	58%	5	56%	12	46%
	Parent-infant interaction	0	0%	2	17%	2	22%	4	15%
	Emotional / mental health support	4	80%	8	67%	8	89%	20	77%
Intervention formats^a^	Home visits	0	0%	4	33%	3	33%	7	27%
	Mobile communication (phone calls / apps)	4	80%	5	42%	0	0%	9	35%
	Individual psychotherapy	0	0%	2	17%	2	22%	4	15%
	Support groups / group therapy	1	20%	3	25%	5	56%	9	35%
Intervention facilitator^a^	Self-help (manual / app / making time for self)	0	0%	3	25%	1	11%	4	15%
	Peer-support (mothers with similar background)	3	60%	3	25%	4	44%	10	38%
	Professional support (health visitors, psychologists, social workers) Other (music professionals)	1	20%	10	83%	5	56%	16	62%
Comparison group	Treatment as usual (TAU)	5	100%	12	100%	4	44%	21	81%
	Active treatment	0	0%	0	0%	1	11%	1	4%
	Baseline measures (single group)	0	0%	0	0%	4	44%	4	15%
Study arms	One (no parallel control group)	0	0%	0	0%	4	44%	4	15%
	Two (control group: TAU or active treatment)	5	100%	9	75%	4	44%	18	69%
	Three (two active treatment groups + TAU)	0	0%	2	17%	0	0%	2	8%
	Four (three active treatment groups + TAU)	0	0%	1	8%	1	11%	2	8%
Subjective aspect of social relationships	Loneliness	4	80%	0	0%	0	0%	4	31%
	Social support satisfaction	0	0%	12	100%	9	100%	21	81%
	Loneliness and social support satisfaction	1	20%	0	0%	0	0%	1	4%
Mental health outcomes^a^	Depressive symptoms	5	100%	11	92%	8	100%	24	92%
	Anxiety and/or stress	2	40%	5	42%	3	33%	10	38%
Significant differences in loneliness / SSS outcomes	Yes	2	40%	4	33%	1	11%	7	27%
	Compared to control group	2		4		0		6	
	No	3	60%	8	67%	8	89%	19	73%
	Compared to control group	3		8		5		16	
Significant differences in mental health outcomes	Yes	5	100%	1	8%	5	56%	11	42%
	Compared to control group	5		1		3		9	
	No	0	0%	11	92%	4	44%	15	58%
	Compared to control group	0		11		3		14	

^a^ Categories are not mutually exclusive, so percentages will not add up to 100%

#### Studies using an RCT design

Of the 26 studies, 17 were RCTs. As well as capturing mental health outcomes, the majority of these (*n* = 12/17) included SSS as the only eligible social outcome [3, 5, 14, 22, 27, 30, 32, 33, 46, 50–52]. Four trials measured loneliness [2, 17, 18, 42], and one measured both loneliness and SSS [49].

### RCTs assessing mental health and loneliness

The five RCTs assessing mental health and loneliness, of which one also assessed SSS [49], were judged to be of acceptable methodological quality: four trials were rated at low risk of bias, while one raised some concerns about risk of bias (Table 3).

**Table 3:** Risk of bias assessment of RCTs assessing loneliness (RoB2)

Reference	Randomisation process	Deviations from intended interventions	Missing outcome data	Measurement of the outcome	Selection of the reported result	Overall bias judgement
Arakawa [2]	Some concerns	Low	Low	Low	Low	Low
Dennis, [17]	Low	Some concerns	Low	Low	Low	Low
Dennis [17]	Low	Low	Low	Low	Low	Low
Perkins [42]	Low	Low	Low	Low	Some concerns	Low
Shorey [49]	Low	Low	Some concerns	Some concerns	Low	Some concerns

All five of these RCTs were conducted in high-income countries; four sampled postnatal women, whilst one targeted pregnant women [2]. No studies targeted any male population. One RCT assessed a direct intervention for reducing loneliness [42], whilst the other four measured interventions indirectly related to loneliness. These five RCTs evaluated the effectiveness of interventions delivered via mobile or online formats that aimed to provide emotional support and improve mental health, with all compared to routine perinatal care (Table 4). 

**Table 4:** Study characteristics of RCTs assessing loneliness

Reference, location		Sample		Intervention					Outcomes (*starred where validated)			
	Publication type	Characteristics	Total *N* (*N* Interven. group; *N* Control group)	Direct (D) / Indirect (ID) interven.; components	Formats of delivery	Facilitators	Duration and frequency	Comparison group	Subjective aspect of social relationships scale	Significant group difference in loneliness / SSS post intervention	Mental health scales	Significant group differences in mental health post intervention
Arakawa [2], Japan	Academic journal	Mothers, prenatal, > 50% married or partnered	639 (I = 310; C = 329)	ID; consultations around perinatal physical health and childcare, and emotional support	Individual synchronous mobile communication (text messages or phone calls)	Perinatal health professionals	10-min remote consultations on participants’ demand	TAU: access to a website with information on pregnancy and childcare	Japanese 3-item version of UCLA Loneliness Scale*	Yes	Japanese version of the EPDS*	Depression symptoms (yes)
Dennis, [17], Canada	Academic journal	Mothers, postnatal, > 50% vulnerable (mental health)	41 (I = 20; C = 21)	ID; provision of information on perinatal healthcare, and emotional support	Individual synchronous telephone-based communication	Trained peers	Contact frequency was not standardised, mean phone connection: 34 min	TAU: services from public health	UCLA Loneliness Scale*	No	EPDS*	Depression (yes)
Dennis [18], Canada	Academic journal	Mothers, postnatal, all married, > 50% vulnerable (mental health)	701 (I = 349; C = 352)	ID; provision of information on perinatal healthcare, and emotional support	Individual synchronous telephone-based	Trained peers	4 telephone contacts minimum (mean = 8.8)	TAU: services from public health	UCLA Loneliness Scale*	No	EPDS*; STAI*	Depression (yes); Anxiety (no)
Perkins [42], UK	Academic journal	Mothers, postnatal, symptoms of postnatal depression	89 (I = 44; C = 45)	D; promotion of social interactions, parent-infant attachment, emotional support	Synchronous online song-writing group sessions	Professional music workshop leaders	Weekly 60-min group workshop for 6 weeks	TAU: waitlist	UCLA Loneliness Scale*	Yes	EPDS*	Depression (yes)
Shorey [49], Singapore	Academic journal	Mothers, postnatal, > 50% married and vulnerable (mental health)	138 (I = 69; C = 69)	ID; emotional support	Synchronous or asynchronous individual mobile communication (emails, text messages or phone calls)	Trained peers	1-month access to mobile health app	TAU: in-hospital care; medical appointments and breastfeeding hotline numbers.	UCLA Loneliness Scale*; SSS captured using the PSSP scale*	No	EPDS*; STAI*	Depression (yes); Anxiety (no)

**Notes**: Where we note that any characteristics applied to > 50% of sample we have used this as the threshold for describing the predominant characteristics of the sample. Interventions were classified into direct (D) or indirect (ID) depending on whether they directly addressed components aiming to enhance social connectedness, following the framework developed by Mann et al., [35]

Social support measures: UCLA LS = University of California, Los Angeles Loneliness Scale (validated); PSSP scale: Perceived Social Support for Parenting (validated)

Mental health measures: EPDS: Edinburgh Postnatal Depression Scale (validated); STAI: State & Trait Anxiety Inventory (validated)

SES: Socioeconomic status; D: Direct; ID: Indirect; I: Intervention group; C: Control group

All five RCTs measured depression, whilst two also measured anxiety [18, 49]. Of these 5 RCTs, one assessed loneliness as primary outcome [42] whilst the remaining RCTs measured loneliness as secondary outcome. Additionally, one trial captured child-care stress [17], and another measured social connectedness [42].

None of these RCTs conducted formal moderation or mediation analyses to explore the temporal relationship of loneliness/SSS and mental health outcomes, so were unable to convey an understanding of mechanisms. Instead, they reported the findings of group comparisons (using *t*-tests, odds ratios, logistic regression and linear mixed models) for both social and mental health variables.

Two of the five trials found significant differences in mental health and in loneliness scores, favouring the intervention group. One of these evaluated a mHealth service providing pregnant women in Japan with access to interactive synchronous communication with health professionals, reporting a significant reduction in loneliness and depressive symptoms in the intervention group at three months post-delivery [2]. The other trial evaluated a 6-week songwriting workshop for postpartum women in the UK involving 60-minute online synchronous sessions led by music professionals, finding a significant reduction in loneliness and depression scores both post-intervention and at follow-up in the intervention group, as well as a significant improvement in social connectedness at follow-up [42].

These two interventions with evidence of mental health and loneliness improvements [2, 42] were the only ones involving contact with professionals: the mHealth service for Japanese pregnant women involved perinatal health professionals [2] whilst the songwriting workshop was facilitated by music professionals with no health qualifications [42]. The other three trials that found no significant differences in loneliness scores were facilitated by peer volunteers [17, 18, 49].

All five RCTs found significant group differences in depression scores favouring the intervention group at post-randomisation. None of the three trials that assessed anxiety-related variables found significant group differences in anxiety scores [18, 49] or child-care stress [17].

Given the mixed evidence in relation to improvements in loneliness outcomes and depression, formal mediation analyses are needed to explore whether reductions in loneliness (or in depression) mediate observed improvements in depression (or in loneliness respectively), or whether these are mutually reinforcing.

### RCTs assessing mental health and SSS (but not loneliness)

Twelve of the total 17 RCTs measured SSS but not loneliness. Four of the 12 RCTs were judged to have a low risk of bias; five raised some concerns regarding their methodological quality, due to minor issues related to deviations from intended interventions and selection of reported results; whilst three trials were judged to be at high risk of bias (Table 5).

**Table 5:** Risk of bias assessment of RCTs assessing SSS (RoB2)

Reference	Randomisation process	Deviations from intended interventions	Missing outcome data	Measurement of the outcome	Selection of the reported result	Overall Bias judgement
Barlow [3]	Low	Some concerns	Low	Low	Low	Low
Barnet [5]	Low	Some concerns	Some concerns	Low	Some concerns	Some concerns
Cote-Arsenault [14]	Low	Some concerns	Low	Some concerns	Low	Some concerns
Gjerdingen [22]	Some concerns	Some concerns	Low	Low	Some concerns	Some concerns
Jesse [27]	Some concerns	Some concerns	High	Low	High	High
Langer [30]	Low	High	High	Some concerns	Low	High
Lenze [32]	Some concerns	High	Low	Some concerns	Low	High
Lenze [33]	Low	Some concerns	Some concerns	Some concerns	Low	Some concerns
Reid [46]	Low	Some concerns	Low	Some concerns	Some concerns	Some concerns
Shorey [50]	Low	Some concerns	Low	Low	Low	Low
Shorey [52]	Low	Low	Some concerns	Low	Low	Low
Shorey [51]	Low	Low	Some concerns	Low	Low	Low

Participants in all these RCTs were perinatal women, except for three that included perinatal men and women within parental dyads [50–52]. Four trials evaluated interventions delivered during pregnancy only, four during the postnatal period, and four during both periods (Table 6).

**Table 6:** Study characteristics of RCTs assessing social support satisfaction (SSS)

Reference, location	Publication type	Sample		Intervention					Outcomes (*starred where validated)			
		Characteristics	Total *N* (*N* Interven. group; *N* Control group)	Direct (D) / Indirect (I) interven.; components	Formats of delivery	Facilitators	Duration and frequency	Comparison group	Subjective aspect of social relationships scale	Significant group difference in loneliness / SSS post intervention	Mental health scales	Significant group differences in mental health post intervention
Barlow [3], UK	Academic journal	Mothers, postnatal, > 50% vulnerable (SES and mental health)	131 (I = 68; C = 63)	ID; teaching parenting skills and parent-infant attachment	Individual home visits	Professional support (health visitor)	Weekly home visits for 18 months (72 visits were possible but the mean was 41)	TAU (home visits with less frequency, mean = 9 visits)	SSQ*	No	EPDS*; GHQ*	Depression (no), General Mental Wellbeing (no)
Barnet [5], USA	Academic journal	Adolescent mothers, pregnant & postnatal, > 50% vulnerable (SES)	217 (I = 114; C = 103)	ID; teaching parenting skills, provision of emotional support	Individual home visits & group sessions	Peer and professional support (social workers)	Weekly visits of 1.5 h. during pregnancy and 1 year postpartum	TAU provided by school: academics, parenting classes, day care, health care	ASSIS*	No	MHI-5*	Depression (no)
Cote-Arsenault [14], USA	Academic journal	Mothers, Pregnant, with previous perinatal problems	24 (I = 12; C = 11)	ID; promotion of prenatal attachment and psychoeducation for anxiety/depression symptom management	Individual home visits	Professional support (nurse home visitors)	5 home visits during pregnancy and 1 post-birth brief visit	Pregnancy information booklets on the same schedule as the intervention group home visits	SSQ-6*	No	CES-D*; STAI*	Depression (no), Trait and state anxiety (no)
Gjerdingen [22], USA	Academic journal	Mothers, postnatal, all married, > 50% vulnerable (mental health)	39 (I1 Postpartum duola = 12, I2 Peer telephone support = 13; C = 14)	ID; educational, practical and emotional support	Synchronous individual telephone-based communication	Peer and professional support (certified duolas)	Doula: 24 h over 6 weeks; Telephone contacts: frequency not standardised for 3 months	TAU and postpartum depression brochure and resource list	authors’ own measure (unvalidated)	No	CES-D* and PHQ-9*	Depression (no)
Jesse [27], USA	Academic journal	Mothers, Pregnant, > 50% vulnerable (SES)	110 (I = 39; C = 71)	D; Culturally Tailored Cognitive Behavioural Intervention promoting social interactions and emotional/ mental health support	Synchronous group sessions	Professional support (mental health professionals)	Group sessions and weekly homework and 6 sessions of 2 h each	TAU provided by social workers or pregnancy care managers	PPP Social Support Subscale* –	No	EPDS*; PSS*	Antepartum depressive symptoms (no), stress (no)
Langer [30], Latin America (various countries)	Academic journal	Mothers, Pregnant, > 50% vulnerable (SES), previous perinatal problems	2235 (I = 1110; C = 1115)	D; promotion of social interactions, provision of educational, practical and emotional support	Individual home visits	Professional support (social workers or obstetric nurses)	4 home visits at weeks 22, 26, 30 and 34 of pregnancy, max of 6 visits when needed	TAU at antenatal clinics	authors’ own measure (unvalidated)	No	STAI*	Anxiety (no)
Lenze [32], USA	Manuscript	Mothers, Pregnant, > 50% single mothers and vulnerable (SES and mental health)	42 (I = 21; C = 21)	D; psychoeducation on inter-personal skills, and provision of emotional / mental health support	Individual psychotherapy sessions	Professional support (IPT therapists)	8 sessions of IPT	TAU: referred to community resources if needed during telephone assessments	SSQ-12*	Yes	EPDS*	Depression (no)
Lenze [33], USA	Academic journal	Mothers, pregnant & postnatal, > 50% single mothers and vulnerable (SES)	42 (I = 21; C = 21)	D; psychoeducation on inter-personal skills, provision of social connectedness skills and emotional / mental health support	Individual psychotherapy sessions	Professional support (IPT therapists)	8 sessions of IPT	TAU and biweekly contacts for the first 3 months postpartum for assessments	SSQ-12*	No	EPDS*	Depression (no)
Reid [46], UK	Academic journal	Mothers, postnatal	1004 (I1 Self-help manual = 250; I2 Support group = 250; I3 Manual + group = 253; C = 251)	ID; information on parenting skills and provision of emotional support	In-person group sessions	Self-help and Professional support (midwives)	Self-help manual and/or weekly group sessions. 4 groups based on receiving one, none or both interventions	TAU at antenatal clinics	SSQ-6*	No	EPDS*	Depression (no)
Shorey [50], Singapore	Academic journal	Heterosexual couples, postnatal	250 (I = 126, 63 mothers & 63 fathers; C = 124, 62 mothers & 62 fathers)	ID; psychoeducation on postpartum period and parenting skills	Asynchronous online communication via mHealth app (informational resources and discussion forum)	Self-help, peers and health professionals (midwives)	1 month to access mobile health app	TAU: parenting support and postnatal appointments	PSS for Parenting*	Yes	EPDS*	Depression (no)
Shorey [52], Singapore	Academic journal	Heterosexual couples, pregnant & postnatal	236 (I = 59 couples; C = 59 couples)	ID; psychoeducation on postpartum period and parenting skills	Synchronous (phone calls) and asynchronous communication (informational resources and discussion forums)	Self-help, peers and health professionals (midwives)	Two telephone sessions, and 1 month access to mobile health app	TAU: antenatal and postnatal check-ups, optional educational classes	PSS for Parenting*	Yes	EPDS*; STAI*	Depression (yes), Anxiety (yes)
Shorey [51], Singapore	Academic journal	Heterosexual couples, pregnant and postnatal	200 (I = 100; C = 100)	ID; psychoeducation on postpartum period and parenting skills	Synchronous individual or group chats with trained peers, and asynchronous online communication via mHealth app (informational resources and discussion forum)	Self-help, peers and health professionals (midwives)	Access to mobile health app up to 6 months postpartum	TAU: perinatal check-ups, optional antenatal educational classes	PSS for Parenting*	Yes	EPDS*; STAI*	Depression (no), Anxiety (no)

**Notes**: Where we note that any characteristics applied to > 50% of sample we have used this as the threshold for describing the predominant characteristics of the sample. Interventions were classified into direct (D) or indirect (ID) depending on whether they directly addressed components aiming to enhance social connectedness, following the framework developed by Mann et al., [35]

Social support measures: ASSIS: Arizona Social Support Interview Schedule (validated); PPP: Prenatal Psychosocial Profile Social Support Subscale from Support Behaviors Inventory (validated); PSS: Perceived Social Support for parenting (validated but note variability in internal consistency between studies); SSQ: Social Support Questionnaire (validated)

Mental health measures: CES-D: The Center for Epidemiologic Studies Depression Scale (validated); EPDS: Edinburgh Postnatal Depression Scale (validated); GHQ: General Health Questionnaire (validated); MHI-5: short form of the RAND Mental Health Inventory (validated); PHQ-9: Patient Health Questionnaire (validated); PSS: Perceived Stress Scale (validated); STAI: State & Trait Anxiety Inventory (validated)

SES: Socioeconomic status; D: Direct; ID: Indirect; I: Intervention group; C: Control group

Of these 12 RCTs, four evaluated direct interventions for developing interpersonal skills [32, 33] or improving social interactions [27, 30] alongside addressing perinatal mental health. The remaining eight trials assessed indirect interventions in relation to enhancing SSS; these aimed to provide emotional or mental health support, parenting skills training, or support to improve parent-infant attachment.

Five of the 12 RCTs included SSS as primary outcome [14, 22, 30, 32, 50] while seven considered SSS as a secondary outcome [3, 5, 27, 33, 46, 51, 52]. Eleven of the 12 RCTs assessed depressive symptoms as the primary outcome, while one trial assessed anxiety as the primary outcome without assessing depression [30]. Alongside depression, four trials also measured anxiety-related symptoms [14, 27, 51, 52] and general mental wellbeing [3].

Only two of the 12 RCTs in this category described an explicit intention to test a hypothesis about mediation; in both cases to investigate the mediating effect of SSS in improving participants’ mental health [27, 30]. Only one [27] conducted formal mediation analyses to test this hypothesis, whilst the other presented separate models for social and mental health outcomes but no formal mediation analysis [30]. The remaining ten trials presented group comparisons (*t*-tests, analysis of covariance, or mixed models) for social and mental health variables separately.

In a formal mediation analysis, among a sample of 110 pregnant, rural, minority, low-income women at risk for antepartum depression, Jesse et al. [27] found that a Culturally Tailored Cognitive Behavioural Intervention had no significant effect on SSS and that SSS did not mediate a reduction in either of the two depression outcomes measured. While the included paper did not report the effect of the intervention on mental health outcomes, an earlier published analysis of the same (but slightly larger) sample of 146 women reported that those in the intervention group had a significant reduction in past-fortnight depression scores (but not past-week depression scores), but this only applied to the sub-sample of women rated as low-moderate risk for antepartum depression and not to the sub-sample rated as high-risk for antepartum depression [28]. The authors did not present a comparison for the full sample, and these sub-group analyses were underpowered. We inferred from both studies that there was: uncertainty (due to power issues) over whether the intervention was effective at reducing depression scores in pregnant women at low risk of antepartum depression, no evidence that the intervention improved SSS, and no evidence of a mediating effect of SSS improvements on improved antepartum depression [27].

A trial assessing a parenting programme for perinatal heterosexual couples in Singapore was the only RCT of the twelve in this category to evidence significant improvements in both SSS and mental health (depression and anxiety) scores [52]. The Supportive Educational Parenting Program (SEPP) assessed was an indirect intervention focused on parenting skills, whereby perinatal couples received two educational telephone calls from midwives (one prenatal and one postnatal) and one month’s access to a mobile health app (including a discussion forum where asynchronous communication was enabled with other participant couples and midwives). Control couples received routine care and optional educational classes on mother and infant care.

Two other RCTs studied SEPPs for heterosexual couples in Singapore and identified significant improvements in SSS scores but not in mental health outcomes compared to controls [50, 51]. The SEPPs evaluated in these two RCTs included telephone educational calls with midwives or any synchronous communication with healthcare professionals [50, 51]. One of these RCTs evaluated the delivery of SEPP for couples in the postnatal period only [50], whilst the other evaluated delivery in both perinatal periods [51]. Additionally, the latter matched participants with trained volunteers who offered peer support via private or group chats [51].

In addition to the aforementioned Singaporean trials assessing educational parenting programmes [50–52], one other RCT (of the twelve in this category) found a significant increase in SSS scores, but no significant difference in mental health outcomes after the intervention compared to controls [32]. This RCT evaluated a direct intervention comprising eight individual brief Interpersonal Psychotherapy (IPT) sessions aimed at improving interpersonal skills and providing mental health support in a US sample of pregnant women, with the control group receiving routine care [32]. Another RCT of eight brief IPT sessions for pregnant women with similar control group conditions included eight additional IPT-Dyad sessions after delivery but did not find any significant group differences in SSS or depression scores [33].

Apart from the Singaporean trial including telephone educational sessions with midwives [52], no other trials in this category found a significant improvement in mental health outcomes after the intervention compared to control groups. Two of these RCTs provided emotional and practical support via telephone and home visits [22, 30] and three RCTs promoted parenting skills via home visits [3, 5, 14], but none found significant group differences in SSS or in mental health scores post-intervention.

In summary, only one trial in this category presented a formal mediation analysis, which did not support a mediating effect of SSS in improving antenatal depression [27]. Three trials identified significant improvements in SSS but none in mental health, eight trials found no improvements in either SSS or mental health, and one trial provided evidence to support effectiveness in improving both SSS and mental health but did not conduct formal mediation analyses [52] (Table 6). One RCT measured both loneliness (see above) and SSS and found no significant group difference in loneliness, SSS or anxiety scores but identified a significant decrease in depression scores at three months follow-up [49]. Together these studies do not provide evidence to support the hypothesis that SSS mediates improvements in mental health. These findings should be interpreted with caution, as the certainty of the evidence is hampered by the use of vote-counting methods rather than meta-analysis, which was precluded given substantial heterogeneity.

#### Studies using a non-RCT design

The nine non-RCT studies were considered to be at moderate (*n* = 4), serious (*n* = 3), or critical (*n* = 2) risk of bias (Table 7). These studies included four quasi-RCTs, four single-group designs comparing measures pre- and post-intervention, and one prospective cohort study comparing groups defined by exposure to a self-help intervention (Table 8).

**Table 7:** Risk of bias assessment of non-RCT study designs (ROBINS-I)

Reference	Confounding	Selection of participants	Classification of interventions	Deviations from intended interventions	Missing data	Measurement of outcomes	Selection of the reported result	Overall Risk of bias judgement
Barlow [4]	Serious	Serious	Low	Low	Critical	Serious	Low	Critical
Cassidy [11]	Moderate	Low	Low	Serious	Serious	Low	Low	Serious
Futterman [21]	Moderate	Low	Moderate	Low	Moderate	Low	Low	Moderate
Hung [26]	Critical	Critical	Moderate	Low	Serious	Low	Low	Critical
Lederer, [31]	Serious	Moderate	Low	Moderate	Serious	Moderate	Low	Serious
Lenze [34]	Serious	Low	Low	Moderate	Serious	Low	Low	Serious
Mundell [39]	Moderate	Low	Low	Moderate	Moderate	Moderate	Low	Moderate
Posmontier [45]	Moderate	Low	Moderate	Moderate	Low	Low	Low	Moderate
Woolhouse [60]	Low	Low	Moderate	Moderate	Moderate	Low	Low	Moderate

**Table 8:** Study characteristics of non-RCTs (all assessing SSS only)

Reference, location	Study Design	Publication type	Sample		Intervention						Outcomes (*starred where validated)			
			Characteristics	Total *N* (*N* Intervention group; *N* Control group)	Direct (D) / Indirect (I) interventions; components	Formats of intervention delivery	Intervention facilitators	Intervention duration and frequency	Comparison group	Subjective aspect of social relationships scale		Significant results^a^ in reduction in loneliness / SSS scores	Mental health scales	Significant results^a^ in mental health scores
Barlow [4], UK	One group pre/post comparison	Project report	Mothers, Pregnant & postnatal, > 50% vulnerable (SES and mental health)	123 (only 42 completed SSS outcome measures)	D; promotion of social interactions, teaching parenting skills and provision of emotional support	Individual home visits and synchronous group sessions	Trained peers	Weekly home visits throughout pregnancy and 1 year postpartum	Baseline	MSSI*		Yes	HADS*	Depression (yes), Anxiety (yes)
Cassidy [11], USA	One group pre/post comparison	Academic journal	Mothers, Pregnant & postnatal, > 50% vulnerable (SES and mental health)	20	ID; promotion of parenting skills, parent-infant attachment, emotional /mental health support	Individual home visits and group sessions	Professional support (perinatal health workers)	Medical care, parenting education daily, for 15 months max	Baseline	SSQ*		No	BDI-IA*	Depression (yes)
Lederer, [31], UK	One group pre/post comparison	Project report	Mothers, Pregnant & postnatal, > 50% single mothers and vulnerable (SES)	17	ID; teaching parenting skills, provision of practical and emotional support	Individual home visits and group sessions	Trained peers	Home visits and support groups during pregnancy and 1 year postpartum	Baseline	MSSI*		No	HADS*	Depression (no), Anxiety (no)
Futterman [21], South Africa	Non-randomised controlled trial	Academic journal	HIV+ mothers, Pregnant & postnatal, > 50% single, insecure housing, unemployed)	160 (I = 77; C = 83)	D; promotion of social interactions, provision of informational and emotional support related to perinatal mental health	Individual peer support and group sessions	Trained peers (mentor mothers - the mothers2mothers peer-mentoring program)	Individual peer support & 8 group CBT sessions during pregnancy and postpartum	TAU: standard services provided by midwives and counsellors	MOS-SSS*		No	CES-D*	Depression (yes)
Hung [26], Taiwan	Non-randomised controlled trial	Academic journal	Mothers, postnatal, all married	230 (I1 maternal care = 35; I2 infant feeding = 47; I3 newborn care = 29; C = 119)	ID; teaching parenting skills and postnatal healthcare	Group sessions (classes)	Health professionals (nurses)	3 parenting classes: 50 min per topic, 1 day per class	TAU: unspecified	SSA*		No	own scale to capture women’s perception of postpartum stress	Postpartum stress (no)
Lenze [34], USA	One group pre/post comparison	Academic journal	Mothers, Pregnant & postnatal, > 50% vulnerable (SES and mental health)	9	D; psychoeducation on inter-personal skills, promotion of parent-infant attachment and provision of emotional / mental health support	Individual psychotherapy sessions	Professional support (IPT therapists)	7 IPT antenatal weekly sessions and 8 postnatal biweekly sessions	Baseline	SSQ-6*		No	EPDS*	Depression (yes)
Mundell [39], South Africa	Non-randomised controlled trial	Academic journal	HIV+ pregnant mothers, > 50% vulnerable (SES)	279 (I = 129; C = 150)	D; psychosocial support for improving interpersonal skills and mental health	Group sessions	Peers and masters-level psychology students	10 weekly sessions of psychosocial support groups	TAU at antenatal clinics	Multidimensional Social Support Inventory (not validated, scale adapted by authors)		No	CES-D*	Depression (no)
Posmontier [45], USA	Non-randomised controlled trial	Academic journal	Mothers, postnatal, > 50% single mothers and vulnerable (mental health)	61 (I = 41; C = 20)	D; promotion of inter-personal skills and provision of emotional / mental health support	Individual psychotherapy sessions via phone calls	Professional support (IPT certified nurse / midwives)	8 IPT sessions of 50 min each, for 12 weeks max.	TAU: referral to a variety of mental health professionals	SSQ*		No	EPDS*	Depression (yes)
Woolhouse [60], Australia	Cohort study using longitudinal data to simulate potential intervention effects of exposure to self-help	Academic journal	Mothers, postnatal, > 50% married	1507 (I = 730; C = 776)	ID; engagement in activities promoting selfcare / emotional wellbeing	NA (a measure of frequency of time for self – simulating intervention effects by comparing exposure to self-help)	Self help	Timeforself over 6 months; “I”: Once a week or more; “C”: less than once a week / never	Women who reported that had time for themselves (15%)	own scale to capture how satisfied mothers were with the support they received from their partner		No	EPDS*	Depression (no)

^a^ ‘Significant results’ refers to significant group differences post intervention for studies using comparison groups, whilst it refers to significant differences in post-intervention scores compared to baseline scores for single group designs

Note: Where we note that any characteristics applied to > 50% of sample we have used this as the threshold for describing the predominant characteristics of the sample. Interventions were classified into direct (D) or indirect (ID) depending on whether they directly addressed components aiming to enhance social connectedness, following the framework developed by Mann et al., [35]

Social support measures: MOS-SSS: Medical Outcomes Study - Social Support Survey (validated); MSSI Maternal Social Support Index (validated); SSA: Social Support APGAR (validated); SSQ: Social Support Questionnaire (validated)

Mental health measures: BDI: Beck’s Depression Inventory (validated); CES-D: The Center for Epidemiologic Studies Depression Scale (validated); EPDS: Edinburgh Postnatal Depression Scale (validated); HADS: Hospital Anxiety and Depression Scale (validated); SES: Socioeconomic status; D: Direct; ID: Indirect; I: Intervention group; C: Control group

All nine non-RCT studies targeted women as participants: one during pregnancy, three postnatally, and five addressed both perinatal periods. Five of the nine studies assessed direct interventions for promoting interpersonal skills or developing social interactions. Additionally, these five interventions provided emotional support and promoted parenting skills or parent-infant attachment. The other four studies evaluating indirect interventions provided emotional support and/or parenting skills training.

All nine studies assessed depression as primary outcome except one that measured postnatal stress [26]. In addition to depression, two studies measured anxiety [4, 31]. All nine studies measured SSS (eight as a primary outcome), but none measured loneliness. No study conducted formal investigation of SSS as a putative moderator or mediator of the effect of the intervention on mental health outcomes.

Only one of the nine non-RCT studies, an English single-group pre- and post-intervention design, found a significant increase in SSS scores and also identified significant post-intervention improvements in mental health; specifically, anxiety and depression measures [4]. This direct intervention provided home visits by trained volunteers to develop informal support networks and parenting skills.

Four of the nine non-RCT studies found a significant improvement in depression symptoms but not in SSS scores compared to controls or baseline, but none of these assessed any other mental health outcome [11, 21, 34, 45]. Three of these studies evaluated direct psychosocial interventions for developing social interactions or interpersonal skills among pregnant and postnatal women. Of these, one evaluated ‘mentor mothers’ delivering perinatal informational and educational support (related to healthcare, feeding and health promotion) as well as peer-delivered cognitive-behavioural group sessions [21]. Two evaluated health professionals offering individual IPT sessions [34, 45]. The other study that found significant improvement in depression scores (but not SSS) was an indirect intervention aiming to improve maternal psychosocial functioning and enhance infant attachment in a sample of pregnant non-violent offenders in the US [11]. This single-group study compared measures pre- and post-intervention, which involved mental health professionals delivering a programme enabling access to individualised social services and a parenting intervention based on the Circle of Security Perinatal Protocol [13] during pregnancy (whilst participants lived in a treatment facility) and in the postnatal period (when participants lived in a residential facility).

The remaining four non-RCT studies found no evidence to support improvements in mental health or SSS. One evaluated a direct intervention for HIV-positive mothers involving provision of psychosocial support for improving interpersonal skills and mental health [39]. The other three evaluated indirect interventions, including a proxy for self-help [60], nurse-led teaching of parenting skills and postnatal healthcare [26], and peer-delivered teaching of parenting skills and provision of practical and emotional support [31], and none reported significant differences in SSS or mental health outcomes attributable to the intervention.

In summary, only one non-RCT study provided some evidence to support improvements in both SSS and mental health, and none of these studies presented formal mediation analyses. Four studies found improvements in depression symptoms but not in SSS, whilst four studies did not find significant changes in either mental health symptoms or SSS. Together these non-RCT findings suggest that the impacts of interventions on SSS appear to be different to impacts on mental health outcomes. However, our certainty in this is limited, considering the moderate to critical risk of bias and high heterogeneity of non-RCT studies (sample sizes, demographics, intervention components, data analysis methods, outcome measures).

## Discussion

### Main findings

In the first review to synthesise evidence, including identifying any mechanistic findings, on the effectiveness of interventions addressing both mental health and subjective aspects of social relationships in the perinatal period, we did not find evidence to support the hypothesis that addressing loneliness/SSS might be a means of improving mental health in perinatal parents. However, we did find limited evidence that perinatal interventions might improve both (or either of) mental health and social connectedness. Of the 26 studies included in this review, seven provided evidence to support the effectiveness of interventions addressing social outcomes (loneliness or SSS) [2, 4, 32, 42, 50–52], of which four supported effectiveness in improving both loneliness/SSS and mental health symptoms [2, 4, 42, 52]. Notably, almost all those seven interventions involved synchronous contact with professionals providing informational or educational support related to parenting and/or perinatal health. We did not identify any studies of interventions targeting parents after a stillbirth or miscarriage, despite the contribution of bereavement-related stigma to loneliness, impaired SSS and psychological difficulties.

Eleven of the 26 studies in this review provided evidence to support the effectiveness of interventions for improving perinatal mental health (four of which also reported significant improvements in the social outcomes, as reported above) [2, 4, 11, 17, 18, 21, 34, 42, 45, 49, 52]. These interventions provided emotional support facilitated either by trained peers or professionals, but six of these were indirect interventions and did not state a primary aim related to enhancing social connections. Of the eleven direct interventions for enhancing social connectedness, five were not supported by evidence of effectiveness in improving both loneliness/SSS and mental health symptoms.

The trial evidence we identified showed that perinatal mental health interventions found to be effective for alleviating loneliness or improving SSS involved professionals as facilitators, whereas those found to be effective for treating depression involved peer contact. We cannot infer too much from this observed pattern and formal mechanistic evaluation is required, taking into account appropriate confounders, before attributing effects to facilitator type. Our findings do suggest that effective interventions for improving perinatal loneliness and SSS might involve different components (and pathways) to effective interventions for treating perinatal depression. The current evidence does not suggest that improvements in loneliness/SSS necessarily mediate improvements in perinatal mental health or vice versa. However, given limited sample sizes and minimal formal mediation/moderation analyses in this review, further work is needed to test mediation and moderation by each in mechanistic pathways. Overall, only two RCTs described an explicit intention to investigate mechanisms, but only one of these conducted formal mediation analysis, and did not find support for their hypothesis that reductions in SSS mediate reductions in antenatal depression [27]. We therefore know little about mechanisms of change in relation to interventions addressing perinatal social outcomes and perinatal mental health. We recommend that future interventional studies include mediation analyses to understand the links between perinatal social outcomes and perinatal mental health.

In our review only five studies reported effect sizes and only three presented standard error estimates. This precluded us from using quantitative synthesis methods that could offer a more formal assessment of the certainty of evidence collected. We encourage triallists of perinatal interventions to follow PRISMA guidelines and to estimate effect sizes that are comparable with those for similar evaluations, permitting formal methods for combining data and improving precision of estimates.

Our review highlights not only the lack of mechanistic studies but also the relative lack of trials of perinatal mental health interventions that measure loneliness compared to those measuring SSS, and of trials measuring perinatal mental health problems beyond depression and anxiety. Future interventional research on perinatal mental health interventions should specifically target loneliness, given the high estimates of reported loneliness among this population [29] and its role as a potential risk factor for mental ill-health [38]. Such trials should use validated loneliness measures and a broader range of perinatal mental health outcomes.

Only one study [49] measured both loneliness and SSS, but as neither were significantly ameliorated, this limited the degree to which different mechanisms might explain why interventions may influence one and/or the other.

We also identified that among interventional studies measuring perinatal loneliness, only one targeted the antenatal period [2], suggesting the need for more research on the impact of perinatal mental health interventions on loneliness during pregnancy.

### Findings in the context of other studies

A key feature of studies in our review with positive findings in improving depression were delivery of emotional support via online or telephone communication. These findings are consistent with those of a recent umbrella review of studies evaluating psychological interventions for perinatal depression, which concluded that internet-based interventions were time- and cost-effective [7]. They are also consistent with those of a broader review of interventional studies delivering different modes of parental support, which noted promising findings in reducing loneliness for parental interventions involving telehealth, home visiting peer support, parenting programmes, interpersonal skills training, and short-term cognitive therapy [40]. Regarding preferences for formal or informal support, previous survey findings suggest that postpartum women prefer informal to formal sources of support, and that postpartum depression is negatively associated with satisfaction with formal and informal instrumental support and informal psychological support [1]. In the context of those findings, our results suggest a need to design and evaluate interventions maximising formal and informal instrumental support and informal psychological support.

### Strengths and limitations

We used a robust and systematic search strategy that was sufficiently broad to retrieve studies addressing a wide range of mental health symptoms in the perinatal period and different subjective experiences of social relationships of mothers and/or fathers. We did not restrict our search to trials of evaluations in which the stated intention was to address loneliness/SSS, which broadened the pool of studies from which we could draw valid inferences. Our use of independent screening and quality evaluation of all records, conducted by two reviewers, was a strength, as was the clinical and research experience brought by members of our research team.

Nevertheless, despite the search aiming to include studies published in English or Spanish, we only identified eligible studies in English, primarily based in high-income countries. We acknowledge the possibility of omitting some trials published in other languages from low- and middle-income settings. Additionally, samples in included studies were primarily female, and most studies assessing loneliness were focused on the postnatal period. All included studies that presented significant improvements in mental health and/or social outcomes were conducted in high-income countries; participants sampled in the majority of these studies were predominantly living with a partner, and were vulnerable to or experiencing mental health difficulties. These limitations on sample representativeness limit the generalisability of our results.

We also recognise that our grey literature search was limited to eight search engines, and excluded some important grey literature databases, and this may have overlooked important findings. We included studies using unvalidated measures with the aim of broadening the scope of our search, provided that they met our inclusion criteria. Although only five of the included studies used unvalidated measures, this represented a potential measurement bias (highlighted in tables) and should be considered when interpreting findings.

The heterogeneity of studies meant that a meta-analysis could not be conducted, and the low proportion of studies rated at low risk of bias (31%) prompts caution in interpreting our overall findings. Without the option of meta-analysis, results were synthesised using vote counting based on the direction of effect, which limited a formal assessment of the certainty of evidence, but throughout our review we considered differences in study quality, tabulating magnitude of effect sizes for context.

### Clinical, policy and research implications

Considering the unique biological and psychosocial risk factors applied to the perinatal period and the damaging consequences to families of poor mental health, there is a clear potential for perinatal mental health interventions to make an important contribution to social connectedness and vice versa. However, we found that most of the studied interventions that significantly improved mental health symptoms did not appear to alleviate loneliness/SSS. We also found little evidence to suggest that addressing loneliness/SSS can improve perinatal mental health or vice versa, largely due to the lack of formal mediation analyses. We also noted that our searches identified no evaluations of perinatal mental health interventions that included approaches addressing social cognitions in perinatal parents. Given that this type of intervention has been identified as promising for alleviating loneliness in populations of people with mental health problems [35] and in the general population [37], this would be worth developing for the perinatal period.

The heterogeneity in the components of the interventions and the outcomes identified in our review suggest that it may be important to investigate further the following intervention components in the perinatal population: type of synchronous interactions, the content of information provided, the models of emotional support offered, the role of facilitators and the nature of the skills delivered. More mechanistic trials that explicitly evaluate potential mediating effects are needed to evaluate the effectiveness of perinatal mental health interventions, as well as cost-effectiveness studies, so that policy decisions can be made about whether addressing loneliness and SSS might improve perinatal parents’ mental health.

Future research should also evaluate the effectiveness of appropriate interventions in low- and middle-income countries, parents in the antenatal period, single parents, male parents, gender minority groups, and special risk populations (such as parents of preterm infants, parents with psychiatric histories, and those bereaved by miscarriage or stillbirth).

This information will be key for gaining a better understanding of perinatal needs and identifying contexts and populations in which targeting loneliness might be relevant to improving parents’ mental health in the perinatal period. Future research should also examine resource requirements for potential implementations of effective interventions. This would have implications for evidence-based guidelines on perinatal care nationally, with potential benefits for parental wellbeing and childhood psychosocial development.

## Conclusions

Our findings highlight the limited evidence describing the effectiveness of interventions for improving mental health during the perinatal period that also alleviate loneliness/SSS, and specifically the lack of mechanistic studies delineating these pathways. We did not find evidence that addressing loneliness/SSS can improve perinatal mental health or vice versa, due to the lack of studies investigating mechanisms formally. There is a clear need for further mechanistic evaluations of interventions that address subjective aspects of social relationships as well as preventing or treating a broad range of mental health outcomes perinatally, particularly where intervention occurs early on (i.e. in pregnancy). This systematic review identifies clear evidence gaps, focussing interest on areas for future work by those who develop and trial perinatal mental health interventions.

## Electronic supplementary material

Below is the link to the electronic supplementary material.

Supplementary Material 1

## Data Availability

The datasets used and/or analysed during the current study are available from the corresponding author on reasonable request.

## Abbreviations

IPT: Interpersonal Psychotherapy
PRISMA: Preferred Reporting Items for Systematic Reviews and Meta-Analyses
RCT: Randomised controlled trial
SSS: Social support satisfaction
SEPP: Supportive Educational Parenting Program

## References

[1] Ando H, Shen J, Morishige KI, Suto S, Nakashima T, Furui T, Kawasaki Y, Watanabe H, Saijo T. Association between postpartum depression and social support satisfaction levels at four months after childbirth. Arch Psychiatr Nurs. 2021;35(4):341–6. 10.1016/j.apnu.2021.03.010. 34176574 10.1016/j.apnu.2021.03.010

[2] Arakawa Y, Haseda M, Inoue K, Nishioka D, Kino S, Nishi D, Hashimoto H, Kondo N. Effectiveness of mHealth consultation services for preventing postpartum depressive symptoms: a randomized clinical trial. BMC Med. 2023;21(1):221. 10.1186/s12916-023-02918-3 *. 10.1186/s12916-023-02918-3 PMC10294407 37365535

[3] Barlow J, Davis H, McIntosh E, Jarrett P, Mockford C, Stewart-Brown S. Role of home visiting in improving parenting and health in families at risk of abuse and neglect: results of a multicentre randomised controlled trial and economic evaluation. Arch Dis Child. 2007;92(3):229–33. 10.1136/adc.2006.095117 *. 10.1136/adc.2006.095117 PMC2083433 17068074

[4] Barlow J, Coe C. (2012) Family Action Perinatal Support Project Research Findings Report. Warwick Medical School. Retrieved from https://www.family-action.org.uk/content/uploads/2014/06/Perinatal-Support-Project-evaluation-2012-Professor-Jane-Barlow.pdf *.

[5] Barnet B, Duggan K, Devoe M, Burrell L. The Effect of Volunteer Home Visitation for Adolescent Mothers on Parenting and Mental Health Outcomes. Arch Pediatr Adolesc Med. 2002;156(12):1216–22. *. 12444833 10.1001/archpedi.156.12.1216

[6] Bedaso A, Adams J, Peng W, et al. The relationship between social support and mental health problems during pregnancy: a systematic review and meta-analysis. Reproductive Health. 2021;18(162). 10.1186/s12978-021-01209-5. 10.1186/s12978-021-01209-5 PMC8320195 34321040

[7] Branquinho M, Rodriguez-Muñoz MF, Maia BR, Marques M, Matos M, Osma J, Moreno-Peral P, Conejo-Cerón S, Fonseca A, Vousoura E. Effectiveness of psychological interventions in the treatment of perinatal depression: A systematic review of systematic reviews and meta-analyses. J Affect Disord. 2021;291:294–306. 10.1016/j.jad.2021.05.010. 34062397 10.1016/j.jad.2021.05.010

[8] Bristol Medical School: Risk of Bias Info. (2016) ROBINS-I tool. Retrieved January 2021 from https://www.riskofbias.info/welcome/home/current-version-of-robins-i/robins-i-tool-2016

[9] Bristol Medical School: Risk of Bias Info. (2019) Current version of RoB2: Cribsheet summarizing the tool. Retrieved January 2021 from https://www.riskofbias.info/welcome/rob-2-0-tool/current-version-of-rob-2

[10] Campbell M, McKenzie JE, Sowden A, Katikireddi SV, Brennan SE, Ellis S et al. (2020). Synthesis without meta-analysis (SWiM) in systematic reviews: reporting guideline. BMJ; 368:l6890 10.1136/bmj.l6890 10.1136/bmj.l6890 PMC7190266 31948937

[11] Cassidy J, Ziv Y, Stupica B, Sherman LJ, Butler H, et al. Enhancing attachment security in the infants of women in a jail-diversion program. Attach Hum Dev. 2010;12(4):333–53. 10.1080/14616730903416955 *. 10.1080/14616730903416955 20582844

[12] Cohen S, Wills TA. Stress, social support, and the buffering hypothesis. Psychol Bull. 1985;98(2):310–57. 10.1037/0033-2909.98.2.310. 3901065

[13] Cooper G, Hoffman KT, Powell B. The Circle of Security Perinatal Protocol. Unpublished manuscript, Marycliff Institute; 2003.

[14] Cote-Arsenault D, Schwartz K, Krowchuk H, McCoy TP. Evidence-based Intervention with Women Pregnant after Perinatal Loss. MCN. Am J Maternal Child Nurs. 2014;39(3):177–86. *. 10.1097/NMC.0000000000000024 24472794

[15] Cox JL, Holden JM, Sagovsky R. Detection of postnatal depression: Development of the 10-item Edinburgh Postnatal Depression Scale. Br J Psychiatry. 1987;150:782–6. 3651732 10.1192/bjp.150.6.782

[16] Dadi AF, Miller ER, Bisetegn TA, Mwanri L. Global burden of antenatal depression and its association with adverse birth outcomes: an umbrella review. BMC Public Health. 2020;20(1):173. 10.1186/s12889-020-8293-9. 32019560 10.1186/s12889-020-8293-9 PMC7001252

[17] Dennis CL. The Effect of Peer Support on Postpartum Depression: A Pilot Randomized Controlled Trial. Can J Psychiatry. 2003;48(2):115–24. *. 12655910 10.1177/070674370304800209

[18] Dennis CL, Hodnett E, Kenton L, et al. Effect of peer support on prevention of postnatal depression among high risk women: multisite randomised controlled trial. BMJ. 2009;338:a3064. 10.1136/bmj.a3064 *. 10.1136/bmj.a3064 PMC2628301 19147637

[19] Egger M, Smith GD, Schneider M, Minder C. Bias in meta-analysis detected by a simple, graphical test. BMJ. 1997;315(7109):629–34. 9310563 10.1136/bmj.315.7109.629 PMC2127453

[20] Fawcett EJ, Fairbrother N, Cox ML, et al. The prevalence of anxiety disorders during pregnancy and the postpartum period: a multivariate Bayesian meta-analysis. J Clin Psychiatry. 2019;80:18r12527. 31347796 10.4088/JCP.18r12527 PMC6839961

[21] Futterman D, Shea J, Besser M, Stafford S, et al. Mamekhaya: a pilot study combining a cognitive-behavioral intervention and mentor mothers with PMTCT services in South Africa. AIDS Care. 2010;22(9):1093–100. 10.1080/09540121003600352 *. 10.1080/09540121003600352 PMC4019405 20824562

[22] Gjerdingen DK, McGovern P, Pratt R, Johnson L, Crow S. Postpartum doula and peer telephone support for postpartum depression: A pilot randomized controlled trial. J Prim Care Community Health. 2013;4(1):36–43. 10.1177/2150131912451598 *. 10.1177/2150131912451598 23799688

[23] Haddaway NR, Pritchard CC, McGuinness LA. (2021) PRISMA2020: R package and ShinyApp for producing PRISMA 2020 compliant flow diagrams (Version 0.0.2). In Zenodo. 10.5281/zenodo.5082518

[24] Howard LM, Khalifeh H. Perinatal mental health: a review of progress and challenges. World psychiatry: official J World Psychiatric Association (WPA). 2020;19(3):313–27. 10.1002/wps.20769. 10.1002/wps.20769 PMC7491613 32931106

[25] Howard LM, Molyneaux E, Dennis CL, Rochat T, Stein A, Milgrom J. Non-psychotic mental disorders in the perinatal period. Lancet. 2014;384(9956):1775–88. 10.1016/s0140-6736(14)61276-9. 25455248 10.1016/S0140-6736(14)61276-9

[26] Hung CH, Tseng YF, Cheng HR. Effect of group teaching projects on women’s stress during postpartum hospitalization. Kaohsiung J Med Sci. 1995;11:205–2012. *. 7602655

[27] Jesse DE, Bian H, Banks EC, Gaynes BN, Hollon SD, Newton ER. Role of Mediators in Reducing Antepartum Depressive Symptoms in Rural Low-Income Women Receiving a Culturally Tailored Cognitive Behavioral Intervention. Issues Ment Health Nurs. 2016;37(11):811–9. 10.1080/01612840.2016.1229821. 10.1080/01612840.2016.1229821 PMC5198893 27740883

[28] Jesse DE, Gaynes BN, Feldhousen EB, Newton ER, Bunch S, Hollon SD. Performance of a Culturally Tailored Cognitive-Behavioral Intervention Integrated in a Public Health Setting to Reduce Risk of Antepartum Depression: A Randomized Controlled Trial. J Midwifery Womens Health. 2015;60(5):578–92. 10.1111/jmwh.12308. 26261095 10.1111/jmwh.12308 PMC4775081

[29] Kent-Marvick J, Simonsen S, Pentecost R, Taylor E, McFarland MM. Loneliness in pregnant and postpartum people and parents of children aged 5 years or younger: a scoping review. Syst Reviews. 2022;11(1):196. 10.1186/s13643-022-02065-5. 10.1186/s13643-022-02065-5 PMC9451126 36071448

[30] Langer A, Farnot U, Garcia C, Barros F, Victora C, Belizan JM, Villar J. The Latin American Trial of Psychosocial support during pregnancy: effects on mother’s wellbeing and satisfaction. Soc Sci Med. 1996;42(11):1589–97. *. 8771642 10.1016/0277-9536(95)00262-6

[31] Lederer J. (2009) Perinatal Support Project Evaluation Report. *Family Action Southwark Newpin*. https://www.family-action.org.uk/content/uploads/2014/07/Southwark-Perinatal-Support-Project-Evaluation-2009.pdf *.

[32] Lenze SN, Potts MA. Brief Interpersonal Psychotherapy for depression during pregnancy in a low-income population: A randomized controlled trial. J Affect Disord. 2017;210:151–7. 10.1016/j.jad.2016.12.029 *. 10.1016/j.jad.2016.12.029 PMC5292056 28038377

[33] Lenze SN, Potts MA, Rodgers J, Luby J. (2020) Lessons learned from a pilot randomized controlled trial of dyadic interpersonal psychotherapy for perinatal depression in a low-income population. *J Affect Disord, 271*, 286–292. 10.1016/j.jad.2020.03.084 *. 10.1016/j.jad.2020.03.084 PMC7365269 32479328

[34] Lenze SN, Rodgers J, Luby J. A pilot, exploratory report on dyadic interpersonal psychotherapy for perinatal depression. Arch Womens Ment Health. 2015;18(3):485–91. 10.1007/s00737-015-0503-6*. 10.1007/s00737-015-0503-6 PMC4439306 25604869

[35] Mann F, Bone JK, Lloyd-Evans B, Frerichs J, Pinfold V, Ma R, Wang J, Johnson S. A life less lonely: the state of the art in interventions to reduce loneliness in people with mental health problems. Soc Psychiatry Psychiatr Epidemiol. 2017;52(6):627–38. 10.1007/s00127-017-1392-y. 28528389 10.1007/s00127-017-1392-y PMC5487590

[36] Mann F, Wang J, Pearce E, Ma R, Schleif M, Lloyd-Evans B, Johnson S. Loneliness and the onset of new mental health problems in the general population: a systematic review. Soc Psychiatry Psychiatr Epidemiol. 2022;57:2161–78. 10.1007/s00127-022-02261-7. 35583561 10.1007/s00127-022-02261-7 PMC9636084

[37] Masi CM, Chen HY, Hawkley LC, Cacioppo JT. A meta-analysis of interventions to reduce loneliness. Pers Soc Psychol Rev. 2011;15(3):219–66. 10.1177/1088868310377394. 20716644 10.1177/1088868310377394 PMC3865701

[38] Milgrom J, Hirshler Y, Reece J, Holt C, Gemmill AW. Social Support-A Protective Factor for Depressed Perinatal Women? Int J Environ Res Public Health. 2019;16(8). 10.3390/ijerph16081426. 10.3390/ijerph16081426 PMC6518117 31010090

[39] Mundell JP, Visser MJ, Makin JD, Kershaw TS, Forsyth BW, Jeffery B, Sikkema KJ. The impact of structured support groups for pregnant South African women recently diagnosed HIV positive. Women Health. 2011;51(6):546–65. 10.1080/03630242.2011.606356*. 10.1080/03630242.2011.606356 PMC4017076 21973110

[40] Nowland R, Thomson G, McNally L, Smith T, Whittaker K. Experiencing loneliness in parenthood: a scoping review. Perspect Public Health. 2021;141(4):214–25. 10.1177/17579139211018243. 34286652 10.1177/17579139211018243 PMC8580382

[41] Peplau LA. (1985). Loneliness Research: Basic Concepts and Findings. In: Sarason, I.G., Sarason, B.R, editors Social Support: Theory, Research and Applications. *NATO ASI Series, vol 24*. Springer, Dordrecht. 10.1007/978-94-009-5115-0_15

[42] Perkins R, Spiro N, Waddell G. Online songwriting reduces loneliness and postnatal depression and enhances social connectedness in women with young babies: randomised controlled trial. Public Health. 2023;220:72–9. 10.1016/j.puhe.2023.04.017. 37270855 10.1016/j.puhe.2023.04.017

[43] Philpott LF, Savage E, FitzGerald S, Leahy-Warren P. Anxiety in fathers in the perinatal period: A systematic review. Midwifery 2019 Sep. 2019;76:54–101. 10.1016/j.midw.2019.05.013. 10.1016/j.midw.2019.05.013 31176080

[44] Popay J, Roberts H, Sowden A, Petticrew M, et al. Guidance on the Conduct of Narrative Synthesis in Systematic Reviews. ESRC Methods Programme; 2006.

[45] Posmontier B, Neugebauer R, Stuart S, Chittams J, Shaughnessy R. Telephone-Administered Interpersonal Psychotherapy by Nurse-Midwives for Postpartum Depression. J Midwifery Womens Health. 2016;61(4):456–66. 10.1111/jmwh.12411*. 10.1111/jmwh.12411 26970401

[46] Reid M, Glazener C, Murray GD, Taylor GS. A two-centred pragmatic randomised controlled trial of two interventions of postnatal support. BJOG: Int J Obstet Gynecol. 2002;109:1164–70. *. 10.1111/j.1471-0528.2002.01306.x 12387471

[47] Russell DW. UCLA Loneliness Scale (Version 3): reliability, validity, and factor structure. J Pers Assess. 1996;66(1):20–40. 10.1207/s15327752jpa6601_2. 8576833 10.1207/s15327752jpa6601_2

[48] Sarason IG, Levine HM, Basham RB, Sarason BR. Assessing social support: The Social Support Questionnaire. J Personal Soc Psychol. 1983;44(1):127–39. 10.1037/0022-3514.44.1.127.

[49] Shorey S, Chee CYI, Ng ED, Lau Y, Dennis CL, Chan YH. Evaluation of a Technology-Based Peer-Support Intervention Program for Preventing Postnatal Depression (Part 1): Randomized Controlled Trial. J Med Internet Res. 2019a;21(8):e12410. 10.2196/12410*. 10.2196/12410 PMC6744221 31469084

[50] Shorey S, Lau Y, Dennis CL, Chan YS, Tam WWS, Chan YH. A randomized-controlled trial to examine the effectiveness of the ‘Home-but not Alone’ mobile-health application educational programme on parental outcomes. J Adv Nurs. 2017;73(9):2103–17. 10.1111/jan.13293*. 10.1111/jan.13293 28276086

[51] Shorey S, Law E, Thilagamangai, Mathews J, Lim S, Shi L, Chua J, Du R, Chan Y, Tan T, Chee C, Chong Y. Evaluating the Effectiveness of the Supportive Parenting App on Parental Outcomes: Randomized Controlled Trial. J Med Internet Res. 2023;25:e41859. 10.2196/41859. 36645699 10.2196/41859 PMC9887516

[52] Shorey S, Ng YPM, Ng ED, Siew AL, Morelius E, Yoong J, Gandhi M. Effectiveness of a Technology-Based Supportive Educational Parenting Program on Parental Outcomes (Part 1): Randomized Controlled Trial. J Med Internet Res. 2019b;21(2):e10816. 10.2196/10816*. 10.2196/10816 PMC6391716 30758289

[53] Sterne JAC, Hernán M, Reeves B, Savović J, et al. ROBINS-I: a tool for assessing risk of bias in non-randomized studies of interventions. BMJ. 2016;355:I4919. 27733354 10.1136/bmj.i4919 PMC5062054

[54] Sterne JAC, Savović J, Page MJ, Elbers RG, et al. RoB 2: a revised tool for assessing risk of bias in randomised trials. BMJ. 2019;366:I4898. 10.1136/bmj.l4898 31462531

[55] Sterne JAC, Sutton AJ, Ioannidis JPA, Terrin N, Jones DR, Lau J et al. (2011) Recommendations for examining and interpreting funnel plot asymmetry in meta-analyses of randomised controlled trials *BMJ, 343*: d4002 10.1136/bmj.d4002 10.1136/bmj.d4002 21784880

[56] Surkalim DL, Luo M, Eres R, Gebel K, van Buskirk J, Bauman A et al. (2022). The prevalence of loneliness across 113 countries: systematic review and meta-analysis. *BMJ 2022; 376*:e067068. 10.1136/bmj-2021-067068 10.1136/bmj-2021-067068 PMC8826180 35140066

[57] Tebeka R, Yann LS, De Premorel Higgons A, et al. Prevalence and incidence of postpartum depression and environmental factors: The IGEDEPP cohort. J Psychiatr Res. 2021;138:366–74. 10.1016/j.jpsychires.2021.04.004. ISSN 0022-3956. 33932643 10.1016/j.jpsychires.2021.04.004

[58] Wang J, Lloyd-Evans B, Giacco D, Forsyth R, Nebo C, Mann F, Johnson S. Social isolation in mental health: a conceptual and methodological review. Soc Psychiatry Psychiatr Epidemiol. 2017;52(12):1451–61. 10.1007/s00127-017-1446-1. 29080941 10.1007/s00127-017-1446-1 PMC5702385

[59] Wesseloo R, Kamperman AM, Munk-Olsen T, Pop VJ, Kushner SA, and Bergink V. Risk of Postpartum Relapse in Bipolar Disorder and Postpartum Psychosis: A Systematic Review and Meta-Analysis. Am J Psychiatry. 2016;173(2):117–27. 10.1176/appi.ajp.2015.15010124. 26514657 10.1176/appi.ajp.2015.15010124

[60] Woolhouse H, Small R, Miller K, Brown S. Frequency of Time for Self Is a Significant Predictor of Postnatal Depressive Symptoms: Results from a Prospective Pregnancy Cohort Study. Birth Issues Perinat Care. 2016;43(1):58–67. *. 10.1111/birt.12210 26678360

