# Supplementary material for: Impact of interventions addressing perinatal mental health on loneliness and/or satisfaction with social support: systematic review
Source: BMC Psychiatry. 2026 Apr 11;26:383. doi: 10.1186/s12888-026-08035-8 (PMC13159232; doi:10.1186/s12888-026-08035-8)
Supplement: Supplementary file 1 — Supplementary Material 1 [file 12888_2026_8035_MOESM1_ESM.docx]

**Supplementary Material 1. Search strategy**

The electronic database search was conducted on 10th May 2021 (and updated on 18th September 2023). OVID interface was used for searching in Embase (from 1974 to 18/09/2023), APA PsychInfo (from 1806 to 18/09/2023), Maternity & Infant Care Database (from 1971 to 18/09/2023), and Medline® All (from 1946 to 18/09/2023). Additionally, Cochrane Central Register of Controlled Trials and Web of Science Core Collection were consulted. The search was limited to studies of humans and English or Spanish language. No restrictions related to the year of publication were set. Free text terms and MeSH terms, such as “perinatal”, “mental* health”, “interven*” and “lonel*” were used for each of these databases following the PICOS approach and connected by Boolean operators (“OR” for connecting words within a concept, “AND” for connecting concepts). Supplementary Table A.1 presents the detailed list of all used search terms.

Grey literature was searched on 5th July 2021, combining some of the search terms listed in Supplementary Table A.1 (e.g., “perinatal mental health interventions”, “pregnant interventions”, “perinatal programme mental health”) to search eight information sources: Ethos, OpenGrey, Google and five mental health and perinatal health organisations’ websites (Mind, Action on Postpartum psychosis, Home Start, National Childbirth Trust - NCT and Family Action). The results retrieved from each source were screened on title and abstract, and the eligible results were imported to EPPI Reviewer.

**Supplementary** **Table A.1. Search terms**

| Concept | Free text | MeSH terms |
| --- | --- | --- |
| Perinatal population | perinatal OR peri-natal OR postpartum OR post-partum OR antenatal OR ante-natal OR prenatal OR pre-natal OR postnatal OR post-natal OR puerper* OR pregnan* OR gestation OR peripartum OR peri-partum OR parturition OR “child* birth” OR “child* bearing” | exp Perinatal Care/ OR perinatal.mp. OR exp Pregnancy/ OR pregnancy.mp. OR exp Peripartum Period/ OR peripartum.mp. OR exp Prenatal Care/ OR prenatal.mp. OR exp Postpartum Period/ OR post-partum.mp. |
| Mental health conditions | "mental* health*" OR "mental disorder*" OR "mental* ill*" OR "mood disorder*" OR psychiatr* OR depress* OR anxi* OR "bipolar disorder" OR bipolar OR "post traumatic stress" OR "post?traumatic stress" OR schizo* OR psychosis OR "eating disorder*" OR anorexi* OR bulimi* OR "personality disorder*" OR "obsessive compulsive" OR "bab* blue*" | exp Puerperal Disorders/ OR puerperal disorders.mp. OR mental health.mp. OR exp Mental Health/ OR psychiatry.mp. OR exp Psychiatry/ OR mental disorder.mp. OR exp Mental Disorders/ OR exp Depression/ OR exp Suicidal Ideation/ OR exp Cognition Disorders/ OR exp Self-Injurious Behavior/ OR exp Borderline Personality Disorder/ OR exp Mood Disorders/ OR exp Anxiety Disorders/ OR exp Depression, Postpartum/ OR postpartum depression.mp. |
| Interventions | interven* OR treat* OR therap* OR program* OR trial OR prevent* | intervention.mp. or exp Early Intervention, Educational/ or exp Crisis Intervention/ or exp Internet-Based Intervention/ or exp Early Medical Intervention/ or exp Psychosocial Intervention/ |
| Loneliness | Lonel* OR (social* ADJ2 (isolat* OR network* OR support OR contact* OR capital OR connect* OR engag* OR disconnect* OR interact* OR satisf*)) OR (social ADJ3 (subjective OR perceived OR quality)) OR (relation* ADJ2 (confiding OR supportive OR trust)) | loneliness.mp. or exp Loneliness/ OR perceived social support.mp. or exp Social Support/ OR exp Social Isolation/ OR social isolation.mp. |

**Supplementary Table 2. Reported estimates used to conduct Egger’s test to assess publication bias**

| **Study name** | **Outcome measure** | **Type of effect measure reported** | **Number in treatment group** | **Number in control group** | **Effect size estimate** | **Standard error** |
| --- | --- | --- | --- | --- | --- | --- |
| Arakawa (2023) | Loneliness (UCLA 3 item measure) | Mean difference (p value) using linear regression models | 310 | 329 | not given | not given |
| Dennis 2003 | Loneliness (UCLA Loneliness Scale) | Standardized measure of the difference between two group means using t-test; p-value not provided, study reported no statistically significant difference | 20 | 21 | not given | not given |
| Dennis 2009 | Loneliness (UCLA Loneliness Scale) | Standardized measure of the difference between two group means using t-test (p-value) | 349 | 352 | not given | not given |
| Perkins (2023) | Loneliness (UCLA 3 item measure) | Standardized measure of the difference between two group means using t-test (p-value) | 44 | 45 | not given | not given |
| Shorey (2019a) | Loneliness (UCLA Loneliness Scale) | Mean difference (p value) using linear mixed models | 69 | 69 | not given | not given |
| **Barlow (2007)** | **SSS - Social Support questionnaire (satisfaction)** | **Output of an ANCOVA test** | **68** | **63** | **0.004** | **not given** |
| **Barnet (2002)** | **Arizona Social Support Interview Schedule** | **Unstandardised coefficient** | **114** | **103** | **-1.3** | **1.5** |
| Cote-Arsenault (2014) | Social Support Questionnaire | No effect measure presented | 12 | 11 | not given | not given |
| Gjerdingen (2013) | Own measure of SSS | No effect measure presented; p value- differences estimated by one-way analyses of variance or x2 tests | 25 | 14 | not given | not given |
| Jesse (2016) | Social Support subscale – Support Behaviors Inventory | No effect measure provided (only p-value of 0.49) | 39 | 71 | not given | not given |
| Langer (1996) | Own measure of SSS | No effect measure presented for comparison of means but reported as not significant | 1110 | 1115 | not given | not given |
| Lenze (2017) | Social Support Questionnaire | No effect measure presented | 21 | 21 | not given | not given |
| **Lenze (2020)** | **Social Support Questionnaire** | **Effect size estimate for treatment group** | **21** | **21** | **0.12** | **0.36** |
| Reid (2002) | Social Support Questionnaire | No effect measure presented, solely mean scores at each timepoint per intervention group | 753 | 251 | not given | not given |
| Shorey (2017) | Perceived Social Support for Parenting scale | Adjusted standardized measure of the difference between two group means using t-test (p-value) | 126 | 124 | not given | not given |
| Shorey (2019b) | Perceived Social Support for Parenting scale | Adjusted difference between the intervention and parental outcomes at each timepoint using OR | 59 | 59 | not given | not given |
| Shorey (2023) | Perceived Social Support for Parenting scale | No effect measure presented, solely mean scores at each timepoint for each group | 100 | 100 | not given | not given |
| Barlow (2012) | SSS - Maternal Social Support Index (MSSI) | Mean difference (p value) using t-test to assess baseline vs post-intervention | 123 | NA | not given | not given |
| Cassidy (2010) | Social Support Questionnaire | Standardized measure of the difference between two group means using t-test | 20 | NA | not given | not given |
| Lederer (2009) | SSS - Maternal Social Support Index (MSSI) | Difference in the N of mothers in "very difficult isolated life" category on the SS Index (below score 15) after the intervention | 17 | NA | not given | not given |
| **Futterman (2010),** | **SSS - Social Support Scale (satisfaction)** | **Estimated effect of intervention RI-linear (intervention*follow-up interaction term)** | 77 | **83** | **-0.62** | **0.6** |
| Hung (1995) | Social Support Scale | No effect measure presented (expressed as PCA finding); t-test estimate (p value) | 111 | 119 | not given | not given |
| Lenze (2015) | Social Support Questionnaire - Revised | No effect measure presented, solely mean scores at each timepoint | 9 | NA | not given | not given |
| **Mundell (2011)** | **Multidimensional Social Support Inventory (MSSI)** | **Random effects regression planned contrast** | **129** | **150** | **0.82** | **not given** |
| Posmontier (2016) | Social Support Questionnaire | Comparison of means - adjusted pairwise comparison (p value) | 41 | 20 | not given | not given |
| Woolhouse (2016) | Authors' own measure of SSS gained from partner | No effect measure presented; adjusted OR for low satisfaction with emotional support | 730 | 776 | not given | not given |

**Note**: Lines in bold indicate the five studies where effect measures were reported in the text, but of these five only three reported a standard error.

**Supplementary Table 3: Synthesis Without Meta-analysis (SWiM) items**

| SWiM reporting item | Item description | Page in manuscript where item is reported |
| --- | --- | --- |
| Methods |  |  |
| 1 Grouping studies for synthesis | 1a) Provide a description of, and rationale for, the groups used in the synthesis (eg, groupings of populations, interventions, outcomes, study design) | 9 |
|  | 1b) Detail and provide rationale for any changes made subsequent to the protocol in the groups used in the synthesis | 9 |
| 2 Describe the standardised metric and transformation methods used | Describe the standardised metric for each outcome. Explain why the metric(s) was chosen and describe any methods used to transform the intervention effects, as reported in the study, to the standardised metric, citing any methodological guidance consulted | 9 |
| 3 Describe the synthesis methods | Describe and justify the methods used to synthesise the effects for each outcome when it was not possible to undertake a meta-analysis of effect estimates | 7 |
| 4 Criteria used to prioritise results for summary and synthesis | Where applicable, provide the criteria used, with supporting justification, to select the particular studies, or a particular study, for the main synthesis or to draw conclusions from the synthesis (eg, based on study design, risk of bias assessments, directness in relation to the review question) | 9 |
| 5 Investigation of heterogeneity in reported effects | State the method(s) used to examine heterogeneity in reported effects when it was not possible to undertake a meta-analysis of effect estimates and its extensions to investigate heterogeneity | 7 |
| 6 Certainty of evidence | Describe the methods used to assess the certainty of the synthesis findings | 8 |
| 7 Data presentation methods | Describe the graphical and tabular methods used to present the effects (eg, tables, forest plots, harvest plots) | 8 |
|  | Specify key study characteristics (eg, study design, risk of bias) used to order the studies, in the text and any tables or graphs, clearly referencing the studies included |  |
| Results |  |  |
| 8 Reporting results | For each comparison and outcome, provide a description of the synthesised findings and the certainty of the findings. Describe the result in language that is consistent with the question the synthesis addresses, and indicate which studies contribute to the synthesis | 11; 15; 17 |
| Discussion |  |  |
| 9 Limitations of the synthesis | Report the limitations of the synthesis methods used and/or the groupings used in the synthesis and how these affect the conclusions that can be drawn in relation to the original review question | 22-23 |

**Supplementary Figure 1: Findings of Egger’s Test for publication bias** (n=3 eligible studies)


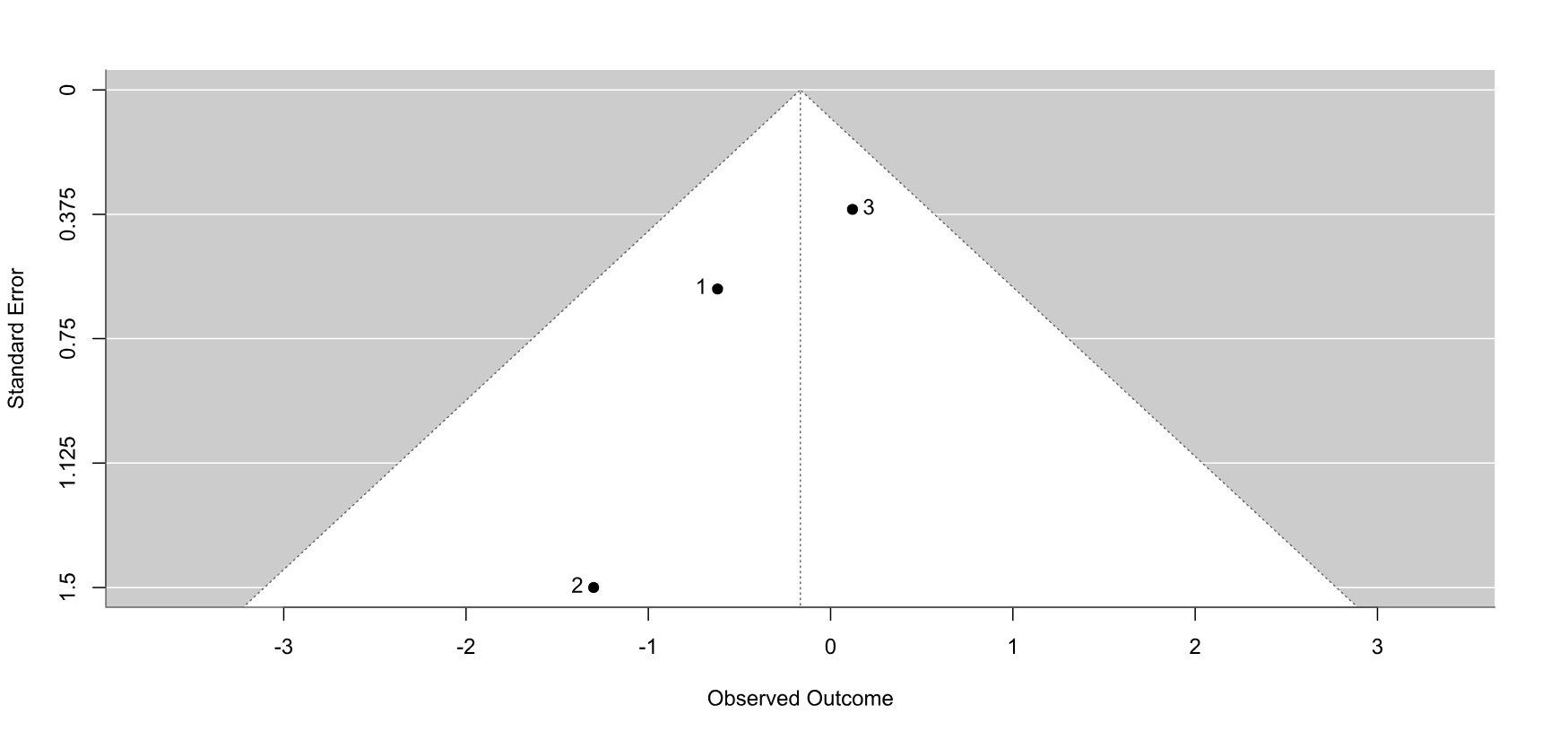


**Note**: Only five of the 26 studies reported effect measures, and of these only three reported a standard error, and could therefore be included in the dataset used for Egger’s test.
